# Towards an Earlier Diagnosis of Acromegaly and Gigantism

**DOI:** 10.3390/jcm10071363

**Published:** 2021-03-26

**Authors:** Jill Sisco, Aart J. van der Lely

**Affiliations:** 1 Acromegaly Community Patient’s Advocacy Organization, Grove, OK 74344, USA; acromegalyinspiration@gmail.com; 2 Division of Endocrinology, Erasmus University MC, 3000 CA Rotterdam, The Netherlands

**Keywords:** acromegaly, pituitary, diagnosis, delay, signs and symptoms

## Abstract

Acromegaly is a rare disease and the clinical features of acromegaly develop insidiously; its diagnosis is often significantly delayed. Therefore, earlier diagnosis will improve the quality of life of the patient and reduce the need for other therapies to control the initial and ongoing damage that acromegaly presents. In this chapter, we describe the view of the patient and the clinician on the importance of earlier diagnosis, as well as on what can be done to speed up this process. Earlier diagnosis will not only improve quality of life and the burden of disease in acromegaly patients, but it will also have a positive impact in the economic burden of this rare disease.

## 1. Introduction

Acromegaly is a rare disease, most often caused by a growth hormone (GH) producing tumor of the anterior pituitary [1]. As the clinical features of acromegaly develop insidiously, its diagnosis is often significantly delayed. Early diagnosis and proper treatment of the diseases can prevent the development of irreversible complications of the disease and improve the quality of life in patients suffering from the disease [2]. Moreover, in the studied cohorts of long-term, well-controlled acromegalic patients, the prevalence and progression of VFs was high, showing that the deleterious effects of GH and IGF-1 excess on bone persist despite achievement of longstanding remission. [3,4,5].

Nguengang Wakap et al. used the publicly available epidemiological data in the Orphanet database to calculate a prevalence estimate of rare diseases [6]. Global point prevalence was calculated using rare disease prevalence data for predefined geographic regions from the "Orphanet Epidemiological file" (http://www.orphadata.org/cgi-bin/epidemio.html accessed on 6 April 2021). Of the 6026 diseases defined by point prevalence, 84.5% of those analyzed have a point prevalence of <1/1,000,000 [6]. Their analysis yields a conservative, evidence-based estimate for the population prevalence of rare diseases of 3.5–5.9%, which equates to 263–446 million persons affected globally at any point in time [6].

In this chapter, we focus on the clinician’s and the patient’s point of view. We will address the problems on how to shorten the delayed diagnosis of this rare disease with its classical signs, symptoms and phenotype.

## 2. Why an Early Diagnosis Is Important: The Clinician’s Point of View

### 2.1. What Is the Problem

Esposito and co-workers studied the diagnostic delay and its impact on morbidity and mortality in a nationwide cohort of patients with acromegaly in Sweden [7]. In the group of 603 acromegaly patients, the mean diagnostic delay appeared to be around 5.5 years [7]. As expected, the mean number of comorbidities was higher in patients with a longer diagnostic delay, although an increased mortality was only found in patients with the longest diagnostic delay [7]. Apparently, a prolonged diagnostic delay is associated with increased morbidity and mortality.

In a commentary on the Esposito paper [7], Boguszewski reiterated that their results reinforce the importance of shortening the latency period between disease onset, diagnosis and treatment to improve patient outcomes [8]. He discussed strategies to allow early identification of acromegaly among public and health professionals, as internists, primary care clinicians, different specialists and dentists are the first point of contact for most of the patients [8]. The added the motto "you must know it to think of it" as a way to reduce time to diagnosis, which could results in lower rates of morbidity and mortality and might positively impact healthcare costs [8].

Zarool-Hassan et al. tried to identify acromegaly features that were most prevalent to promote increased awareness about the disease by healthcare providers in New Zealand [9]. The main pre-diagnosis physical changes participants reported were acral changes, alterations in facial features and oral symptoms. For some, these features were present for more than 10 years before the acromegaly diagnosis [9]. Increased awareness of acromegaly among primary care clinicians as the first-point-of-contact with the healthcare system for most patients seems to be the most important step towards an earlier detection of acromegaly [9].

Whether or not all long-term comorbidities can be prevented with an improved early diagnosis is questionable. Claessen et al., e.g., reported the outcome of a prospective study on the clinical course of arthropathy during follow-up and its relationship to radiographic progression in 58 long-term controlled acromegaly patients [10]. Not surprisingly, the clinical course of arthropathy was indeed related to the radiographic course. On average, hand and lower limb function deteriorated during follow-up, despite large interindividual variations. Joint pain was stable over time. High baseline BMI was a risk factor for functional deterioration in the lower limb. Additionally, in treated acromegaly patients, joint function deteriorates during prolonged follow-up, despite biochemical disease control, although there was interindividual variation [10].

The same author aimed to further characterize cartilage and bone abnormalities associated with acromegalic arthropathy in 26 subjects using MRI [11]. Both in active and controlled acromegaly, structural osteo-arthritis defects were highly prevalent, with thickest cartilage and highest cartilage T2 relaxation times in the active patients. When compared to primary osteo-arthritis subjects, patients with acromegaly seem to have less cysts and bone marrow lesions, but comparable prevalence of osteophytosis and cartilage defects. They concluded that patients with active acromegaly have a high prevalence of structural osteo-arthritis abnormalities in combination with thick joint cartilage. Apparently, patients with acromegaly have a different distribution of structural osteo-arthritis abnormalities visualized by MRI than primary osteo-arthritis subjects, especially of cartilage defects [11].

Another example is the presence of obstructive sleep apnea (OSA), which is a common disorder characterized by upper airway collapse requiring nocturnal ventilatory assistance [12]. Multiple studies have investigated the relationship between acromegaly and OSA, reporting discordant results. Parolin et al. conducted a meta-analysis on the risk for OSA in acromegaly, and to assess the role of disease activity and the effect of treatments [12]. They found that OSA prevalence was similar in patients with active versus inactive acromegaly. In addition, the apnea-hypopnea index (AHI) was similar in active and inactive acromegaly patients. When AHI was compared before and after treatment in patients with acromegaly, a significant improvement was observed after treatment. When AHI was measured longitudinally before and after treatment, a significant improvement was observed after treatment [12].

An important aspect to consider is the effectivity of treatment modalities to controle disease activity. Neggers and co-workers suggested a new concept, called extra-hepatic acromegaly [13]. Medical treatment of acromegaly with somatostatin analogs (LA-SMSA) and the GH receptor antagonist, pegvisomant (PEGV), has made it possible to achieve normal serum IGF1 concentrations in most patients with acromegaly. As they stated, these two compounds, however, impact the GH-IGF1 axis differently, which challenges the traditional biochemical assessment of the therapeutic response. They postulated that LA-SMSA in certain patients normalizes serum IGF1 levels in the presence of still elevated GH actions in extra-hepatic tissues. This may result in persistent disease activity [13]. This concept might explain why certain long-term consequences of the disease despite a normal IGF1 under LA-SMSAs remain progressive.

One of the clinical problems in acromegaly patients is their sleep apnea [14]. Obstructive sleep apnea syndrome (OSAS) is common in active acromegaly and negatively influences quality of life, morbidity, and mortality. Wolters et al. performed a prospective study with 3 predetermined timepoints and a standardized treatment protocol investigates changes in sleep parameters during the first 2.5 years of acromegaly treatment. At baseline, three out of four patients was diagnosed with OSAS [14]. They observed a substantial decrease in prevalence and severity of OSAS following acromegaly treatment, with the largest improvement during the first year. Most patients recover from OSAS following surgical or biochemical control of the acromegaly [14].

Hypertension and other cardiovascular problems are highly prevalent, occurring in more than 40% of patients with acromegaly, and early diagnosis and early aggressive treatment of elevated blood pressure is important irrespective of which acromegaly treatment is employed [15,16,17,18]. The effect of different medical treatments for acromegaly on hypertension and cardiovascular problems is as yet unclear [19,20]. Additionally, on top of that, OSAS, which is as described above present in most patients with acromegaly, exacerbates hypertension [21].

### 2.2. What Can We Do About It

To assist primary care clinicians in this, some years ago a nation-wide program in The Netherlands was organized that existed out of a one-pager that showed the clinical features of acromegaly. This leaflet was sent to all primary care clinicians. They were asked whether some of their patients showed the classical phenotypical features. If so, they were asked to assess IGF-I, and if elevated, call a certain number. This one-pager was sent again 3 months later and again to all primary care clinicians. We hoped and expected to see a temporary increase in new acromegaly patients which unfortunately did not happen (unpublished data).

Caron and co-workers performed an observational, cross-sectional, multicenter non-interventional study in 472 patients at 25 hospital departments in France that treat acromegaly [22]. Multiple correspondence analysis (MCA) was unsuccessful in identifying sign-and-symptom associations at diagnosis. Endocrinologists (29.5% patients) and other clinical specialists (37.2% patients) were commonly first to suspect acromegaly [22]. Morphologic manifestations, snoring syndrome, and asthenia were frequently present at diagnosis, and discrepancy between patient- and physician-reported manifestations were highest for functional signs [22]. The earliest manifestations prior to diagnosis, according to how they were detected, were enlarged hands and feet (functional signs), hypertension (complementary examination) and carpal/cubital tunnel syndrome (functional signs with complementary examination) [22]. Their results confirm the broad range of manifestations at diagnosis and delay in recognizing the disease. Discrepancy rates suggest physicians should obtain the patient’s perspective and seek functional signs during diagnosis [22].

Ohno et l. reviewed records of 81 patients with acromegaly who underwent their first transsphenoidal surgery from 2006 to 2015 at their Niigata Medical and Dental University Hospital [23]. Two groups were compared: those who underwent surgery between 2006 and 2010 (*n* = 35) and those who underwent surgery between 2011 and 2015 (*n* = 46). They compared clinical features and serum GH and IGF-I levels plus hypertension (HT) and diabetes mellitus (DM) prevalence between the two groups. Compared with the early group, microadenomas (<10 mm) were more prevalent in the late group (0% vs. 15.2%, *p* < 0.05). Serum IGF-I SDS score was significantly lower in the late group. In both groups, mean IGF-I SDS was significantly lower in patients without DM than in those with DM. Logistic regression analysis showed that serum GH and IGF-I levels were significantly higher in patients with DM than in those without DM. Apparently, operated cases of GH-producing pituitary adenoma, acromegaly clinical manifestations tended to be milder at diagnosis in later years of the decade, and acromegaly was diagnosed at lower IGF-I levels and in smaller lesions [23].

### 2.3. Is Artificial Intelligence (AI) the Solution?

Already in 2011, Miller et al. discussed the issue that early diagnosis of a number of acromegaly is theoretically possible by the examination of facial photographs [24]. They compared the accuracy of their software program to that of 10 generalist physicians [24]. The reported that the computer model could sort photographs of patients with acromegaly from photographs of normal subjects and was much more accurate than the sorting by practicing generalists [24].

In a recent report by Meng et al., they combined the use of 3D imaging and machine learning techniques in facial feature analysis and identification of acromegalic patients, in an effort to ascertain how both techniques performed in terms of applicability and value in the early detection of the disease in 62 patients with acromegaly and 62 matched controls [25]. Patients were significantly different from normal subjects in many variables, and facial changes of male patients were more significant than female ones. Both male and female patients presented the increase in facial length and breadth, the widening and elevation of the nose, the thickening of vermilion and the enlargement of the mandible [25]. They concluded that 3-dimensional imaging enables comprehensive and accurate quantification of facial characteristics, which makes it a promising technique to investigate facial features of acromegalic patients. This was reconfirmed by several others [26,27].

In a much larger study, Kong et al. developed a new automatic diagnosis and severity-classification model for acromegaly using facial photographs by deep learning on the data of 2148 photographs at different severity levels [28]. Each photograph was given a score reflecting its severity (range 1–3). Their developed model achieved a prediction accuracy of 90.7% on the internal test dataset and outperformed the performance of ten junior internal medicine physicians (89.0%) [28]. The prospect of applying their model to real clinical practices might by promising due to its potential health economic benefits [28].

Wang et al. compared the Receiver operating characteristic (ROC) and discriminant analysis for acromegaly detection by three dimensional facial measurements [29] to explore the difference of detection rate between the two analysis methods. The result shows that the accuracies of three categories from the univariate discriminant analysis, the lateral angles displayed the highest accuracy between all three categories in the female but the lowest rate for the ROC analysis [29].

Park and coworkers studied several popular machine learning algorithms used to train a retrospective development dataset consisting of 527 acromegaly patients and 596 normal subjects [30]. They firstly used OpenCV to detect the face bounding rectangle box, and then cropped and resized it to the same pixel dimensions. From the detected faces, locations of facial landmarks which were the potential clinical indicators were extracted. Frontalization was then adopted to synthesize frontal facing views to improve the performance [30]. Several popular machine learning methods including LM, KNN, SVM, RT, CNN, and EM were used to automatically identify acromegaly from the detected facial photographs, extracted facial landmarks, and synthesized frontal faces. The trained models were evaluated using a separate dataset, of which half were diagnosed as acromegaly by growth hormone suppression test. The best result of their proposed methods showed a positive predictive value of 96%, a negative predictive value of 95%, a sensitivity of 96% and a specificity of 96% [30]. Apparently, modern algorithms based on artificial intelligence can automatically early detect acromegaly with a high sensitivity and specificity.

### 2.4. But What about Privacy

The recognizability of facial images raises patient privacy concerns. In an interesting study by Parks et al., they examined how accurately facial images extracted from computed tomography (CT) scans are objectively matched with corresponding photographs of the 128 scanned individuals [31]. Using facial recognition software, the 2D images of the extracted facial models were compared for matches against five differently sized photo galleries [31]. Their results suggest that the probability of a match between a facial image extracted from a medical scan and a photograph of the individual is moderately high. The facial image data inherent in commonly employed medical imaging modalities may need to consider a potentially identifiable form of “comparable” facial imagery and protected as such under patient privacy legislation [31].

Martinez-Martin stated that we will need to be careful and study of the broader impact of facial recognition technologies (FRT) in health care settings, including issues as liability [32]. The author provided the example that if FRT diagnostic software develops to the point that it is used not just to augment but to replace a physician’s judgment, ethical and legal questions may arise regarding which entity appropriately has liability [32]. Indeed it is of utmost important to assess the relative benefits and burdens of specific FRT uses in health care [32]. Therefore, privacy and data protections are key to advancing FRT and making it helpful [32].

## 3. Why an Early Diagnosis Is Important: The Patient’s Perspective

For a patient living with acromegaly, the cost and burden of delay of diagnosis can be substantial and in many cases irreversible. Not just the monetary factors should be considered, but also the mental, physical, and hormonal tolls that are taken from delays. Monetarily, it is important that they seek surgeons for transsphenoidal surgery with a high success rate. This often leads to higher costs for the patient by seeking surgeons outside of their prospective medical networks. It has also been found, the larger the tumor, the lower the success rate, even with our most experienced surgeons because there are things within the anatomy of the tumor that cannot be aggressively debulked once it invades critical areas. This often leads to the patients being on lifelong specialty medications that are expensive and difficult to manage from both a patient and a clinician perspective. Additionally, if the damage is done during the surgery it typically leads to poor quality of life post-surgery with secondary conditions such as diabetes insipidus or secondary adrenal deficiency. Regarding the mental aspect of living with acromegaly, it must be said that we live in a very visual world. The changes that occur with time in the coarsening of the patient’s features are detrimental to the female and male psyche. The spacing between the teeth, the underbite, the increased hand and foot size, the masculinity of features are difficult, especially for the female patients, which all get worse the longer the diagnosis is delayed. When speaking of a diagnostic delay, how many of the patients are told during the acromegaly search for answers that it is all in our head? The physical changes can affect the joints, with many patients in need for replacements at semi young ages. The cardiovascular system is also affected as many of the patients have enlarged hearts with severe regurgitation. The hormonal effects also lead to decreased sex drives in both male and female patients. Acromegaly is a condition that not only affects the patient, but also the spouse and the entire family. The fatigue that they must deal with is indescribable. They tend to attribute this to every cell in their bodies working harder than it should because it is trying to process the excess GH. In the Patient Focused Drug Development program of the Acromegaly Community Patients Advocacy Organization that took place in January 2021, the top five symptom complaints of acromegaly patients were 1. Fatigue 2. Joint issues 3. Anxiety/depression 4. Headaches 5. Soft Tissue Swelling. They showed that of the patients that took part in the online polling, 76% of patients consider themselves biochemically controlled (Always 22%, Most of the time 35%, Sometimes, 19%) but many do not consider themselves symptomatically controlled (Always 2%, Most of the time 29%, Sometimes 18%). Bottom line, earlier diagnosis and its importance are to improve the patient’s quality of life and reduce the need of other therapies to control the initial, ongoing and irreversible damage that acromegaly presents. We often see that patients diagnosed early do not have the comorbidities or issues that those with delays in diagnosis face. Acromegaly Community patients express that one of their wishes regarding this condition is that they would have been diagnosed sooner in order to have a better outcome. Their patients, more often than not, questioned the changes that were happening to them prior to diagnosis and often contributed these changes to more than aging but could not understand the physical and emotional changes themselves. With treatment, their patients just wanted to feel and look better. Their patients want to help patients of the future; this is why so many are brave enough to post their past and present photos. They have enacted November 1st each year as Acromegaly Awareness Day. On this day many within their community take to social media to share their stories for awareness. They have recently asked if patients would mind submitting their photos for artificial intelligence research, of which their community responded with an overwhelming yes. They do not want their personal information tied to these photos, but they are happy to share if presented in a private, protected way. The Acromegaly online community’s hope is that one day artificial intelligence will be used by any physician that suspects acromegaly and is in a position to, at the very least, refer the patient to a doctor for further testing. They believe such practices as dentists, orthodontists, cardiologists, sleep specialists, gastroenterologists and even unaware endocrinologists could use this technology to aid in the diagnostic process. The Acromegaly online community have found that a physician must know of or suspect acromegaly in order for a proper diagnosis to be made. Diagnosis is key to fighting the debilitating effects of this condition. Delays in diagnosis only prolong suffering and the battle of the patient being in control of this disease instead of the disease being in control of the patient.

## Data Availability

Not applicable.
